# Clinical prediction of intravenous immunoglobulin-resistant Kawasaki disease based on interpretable Transformer model

**DOI:** 10.1371/journal.pone.0327564

**Published:** 2025-07-09

**Authors:** Gahao Chen, Ziwei Yang

**Affiliations:** Department of pediatrics, Affiliated Hospital of North Sichuan Medical College, Nanchong, Sichuan, China; Children’s National Hospital, George Washington University, UNITED STATES OF AMERICA

## Abstract

Intravenous immunoglobulin (IVIG) has been established as the first-line therapy for Kawasaki disease (KD). However, approximately 10%–20% of pediatric patients exhibit IVIG resistance. Current machine learning (ML) models demonstrate suboptimal predictive performance in KD treatment response prediction, primarily due to their limited ability to effectively process categorical variables and interpret tabular clinical data. This study aims to develop and interpretable transformer-based clinical prediction model for IVIG resistant KD and validate its clinical utility. This retrospective study analyzed clinical records of KD patients from the Affiliated Hospital of North Sichuan Medical College (Nanchong, China) between January 1, 2014 and December 31, 2024. A cohort of 1,578 pediatric KD cases was systematically divided into training and validation sets. Six machine learning algorithms - Random Forest (RF), AdaBoost, Light Gradient Boosting Machine (LightGBM), eXtreme Gradient Boosting (XGBoost), Categorical Boosting (CatBoost), and Tabular Prior-data Fitted Network version 2.0 (TabPFN-V2) - were implemented with five-fold cross-validation to optimize model hyperparameters. Model performance was rigorously evaluated using seven metrics: accuracy, precision, recall, F1-score, Matthews correlation coefficient (MCC), area under the receiver operating characteristic (ROC-AUC), and area under the precision-recall curve (PR-AUC). The top-performing model was subsequently subjected to interpretability analysis through Shapley Additive Explanations (SHAP) to elucidate feature contributions. The transformer-based TabPFN-V2 model demonstrated superior predictive performance in KD analysis, achieving an impressive validation set accuracy of 0.97. Comprehensive evaluation metrics confirmed its robust performance: precision 0.98, recall 0.97, F1-score 0.98, MCC 0.95, ROC-AUC 0.99, and PR-AUC 0.99. Global interpretability analysis through kernel SHAP methodology identified the ten most influential predictive features ranked by significance: Coronary artery lesions (CAL), Aspartate aminotransferase (AST), C-reactive protein (CRP), whether it was incomplete KD (KDtype), Neutrophil count (N), Platelet count (PLT), Albumin (ALB), age, White blood cell count (WBC) and Hemoglobin (Hb). Local interpretability analysis revealed distinct correlation patterns with IVIG resistance:AST, CRP, and N demonstrated significant positive correlations, where elevated values corresponded to increased IVIG resistance risk; PLT and ALB showed negative correlations, with higher levels associated with reduced resistance probability. Notably, age and WBC parameters demonstrated threshold effects, where optimal cutoff values enabled re-calibration of single-variable predictive scores. This threshold-dependent relationship suggests potential clinical utility in risk stratification protocols.The TabPFN-V2 model, leveraging an interpretable transformer architecture, demonstrates dual clinical utilities in KD management: (1) accurate prediction of IVIG resistance risk, and (2) data-driven support for personalized therapeutic decision-making. This framework enables probabilistic estimation of treatment resistance likelihood while providing transparent feature contribution analyses essential for developing patient-specific management protocols.

## Introduction

Kawasaki disease (KD), an acute systemic vasculitis predominantly affecting children under five years old, represents the leading cause of acquired pediatric heart disease due to its propensity to induce coronary artery lesions (CAL) [1]. While intravenous immunoglobulin (IVIG) administration remains the established first-line therapy, approximately 10%–20% of patients exhibit IVIG resistance [2], necessitating clinical reassessment of therapeutic strategies. Although retreatment with IVIG is frequently attempted in such cases, concerns persist regarding its therapeutic efficacy, potential adverse effects, and cost-effectiveness [3]. Importantly, IVIG resistance has been strongly associated with an elevated risk of CAL [4]. These clinical realities underscore the critical need to establish robust predictive models capable of early identification of high-risk patients, enabling pediatricians to implement timely therapeutic interventions.

Currently, the prediction of IVIG resistant KD primarily relies on scoring systems and traditional machine learning (ML) model, such as Egami, Kobayashi, and Sano [5]. However, conventional models face significant limitations in processing high-dimensional and large-scale datasets characteristic of IVIG resistant KD, which may compromise both prediction accuracy and clinical applicability [6–8]. These models frequently fail to account for complex interactions among multiple clinical and laboratory variables, potentially leading to unreliable risk stratification. Furthermore, existing ML implementations demonstrate suboptimal performance in handling categorical variables and tabular medical data [9, 10]. Specifically, conventional label encoding methods for categorical variables tend to generate high dimensional sparse matrices, creating computational challenges that hinder effective model training and ultimately result in diminished predictive performance. These limitations underscore the critical need for developing more efficient analytical frameworks specifically optimized for medical tabular data processing, which could substantially enhance both predictive accuracy and clinical decision-making in KD management.

TabPFN presents a novel Transformer-based architecture specifically designed to enhance traditional ML approaches in tabular data processing. The model innovatively implements a bidirectional hierarchical attention mechanism that dynamically learns inter-feature relationships, seamlessly integrating categorical and numerical feature processing through dual-path representation learning. This architecture significantly boosts both computational efficiency and predictive accuracy while maintaining interpretability through its structured attention patterns [11]. The proposed architecture synergizes In-Context Learning (ICL) with Bayesian principles through a probabilistic fusion framework, establishing an adaptive inference mechanism that enables efficient Bayesian integration in deep neural networks. The core innovation of TabPFN lies in its reformulation of posterior approximation as a supervised learning task. Specifically, the model employs a groundbreaking methodological framework in tabular data learning where it: 1) draws function samples from predefined prior distributions, 2) generates synthetic datasets by extracting feature-label pairs, 3) intentionally masks subsets of labels to create missing-value scenarios, and 4) optimizes its parameters to perform Bayesian inference by predicting complete probability distributions conditioned on the observable data patterns. TabPFN-V2 architecture demonstrates substantial advancements through pre-training on a comprehensive 130-million synthetic tabular prediction dataset while achieving state-of-the-art inference efficiency. This next-generation model incorporates optimized computational paradigms specifically engineered for 2025 deployment scenarios.

The opaque nature of ML models may undermine trust among patients and clinicians, consequently restricting their clinical implementation. To address this interpretability challenge, SHapley Additive exPlanations (SHAP) provides game-theoretic principles for quantifying individual feature contributions through optimal Shapley value estimation [12]. As a model-agnostic interpretation framework, Kernel SHAP extends the Local Interpretable Model-agnostic Explanations (LIME) paradigm by operationalizing Shapley values to decompose prediction outcomes into feature-specific attribution scores. This approach enables quantitative assessment of input variable influences across arbitrary ML architectures, offering clinically actionable insights through transparent visualization of feature-prediction interactions. Particularly in medical diagnostics, such interpretability frameworks have demonstrated particular efficacy in elucidating pathological decision pathways, thereby enhancing clinicians’ ability to validate model predictions and optimize therapeutic interventions. Therefore, this study seeks to develop and validate a clinical risk stratification model for IVIG resistant KD through implementation of an interpretable deep learning-based Transformer framework, incorporating state-of-the-art ML methodologies to enhance predictive performance and clinical translatability. This article is presented in accordance with the TRIPOD+AI reporting checklist [13]. For more information, see S1 Table.

## Materials and methods

### Clinical features

This retrospective study analyzed clinical records of KD patients diagnosed at the Affiliated Hospital of North Sichuan Medical College in Nanchong, Sichuan Province, from January 1, 2014 to December 31, 2024. The data were accessed for research purposes in 02/02/2025, after the data collection had been completed. Diagnosis was established according to the 2017 American Heart Association (AHA) guidelines for KD [14]. The final cohort comprised 1,578 confirmed cases, including 170 cases (10.77%) of IVIG resistant KD. Demographic data revealed a male predominance (937 males vs. 641 females; male-to-female ratio 1.5:1), with age at presentation ranging from 0 to 210 months (mean ± SD: 34.10±25.53 months). Incomplete KD was diagnosed when patients presented with 2-3 major clinical features along with compatible laboratory findings, after excluding alternative diagnoses exhibiting exudative conjunctivitis, pharyngeal exudates, ulcerative stomatitis, vesiculobullous rash, generalized lymphadenopathy, or hepatosplenomegaly. In consideration of the potential confounding effects associated with Multisystem Inflammatory Syndrome in Children (MIS-C), individuals with laboratory-confirmed SARS-CoV-2 infection were systematically excluded from the study population [15]. IVIG resistance was defined as either persistent fever (>36 hours) following standard therapy with high-dose IVIG (2 g/kg) and aspirin (30-50 mg/kg/day), or recrudescent fever occurring 2-7 days post-treatment. CAL were identified using echocardiographic criteria (Z-score >2.5 in any coronary segment). The study protocol received ethical approval from the Institutional Review Board of North Sichuan Medical College Affiliated Hospital (approval number: [*2022ER235-1*]). Written informed consent was secured from all participants’ family members prior to study initiation, ensuring compliance with the ethical principles outlined in the Declaration of Helsinki.

### Data collection

The standardized clinical dataset (S1 Appendix) of KD comprises 1,578 cases documenting sex, age at onset, disease classification (complete vs. incomplete KD), coronary artery lesions (CAL) status, recurrence history, and 10 laboratory parameters: C-reactive protein (CRP), erythrocyte sedimentation rate (ESR), white blood cell count (WBC), neutrophil count (N), platelet count (PLT), hemoglobin (Hb), albumin (ALB), total protein (TP), aspartate aminotransferase (AST), and alanine aminotransferase (ALT).

### Statistical analysis

The data preprocessing and analytical procedures were implemented in Python 3.10 utilizing scikit-learn 1.4.2. A comparative evaluation of ML algorithms was conducted, incorporating six distinct ensemble architectures: Random Forest and AdaBoost from scikit-learn, XGBoost 1.7.3, LightGBM 4.1.0, CatBoost 1.2, and TabPFN 2.0. Model performance was rigorously assessed through seven complementary metrics: accuracy, precision, recall, F1-score, Matthews Correlation Coefficient (MCC), along with receiver operating characteristic (ROC) and precision-recall (PR) curves. This study defined true positives (TP), true negatives (TN), false positives (FP), and false negatives (FN) for each predicted class. For instance, for the IVIG resistant class, TP is the number of IVIG resistant samples predicted correctly. TN is the number of non-IVIG resistant samples. FP is the number of IVIG responsiveness predicted as KD. FN is the number of non-IVIG responsive samples.

$$\text{Accuracy}=\frac{TP+TN}{TP+TN+FP+FN}$$ (1)

$$\text{Precision}=\frac{TP}{TP+FP}$$ (2)

$$\text{Recall}=\frac{TP}{TP+FN}$$ (3)

$$\text{F1 score}=2\times \frac{\text{Precision}\times \text{Recall}}{\text{Precision}+\text{Recall}}$$ (4)

MCC represents a statistically robust metric that addresses critical limitations in class-imbalanced learning scenarios. Its $\chi^{2}$-based formulation demonstrates enhanced reliability in both small-sample regimes and skewed class distributions , while simultaneously mitigating stochastic outcome variations through explicit covariance normalization.

$$\text{MCC}=\frac{TP\times TN-FP\times FN}{\sqrt{\left(TP+FP)(TP+FN)(TN+FP)(TN+FN\right)}}$$ (5)

**Fig 1:**
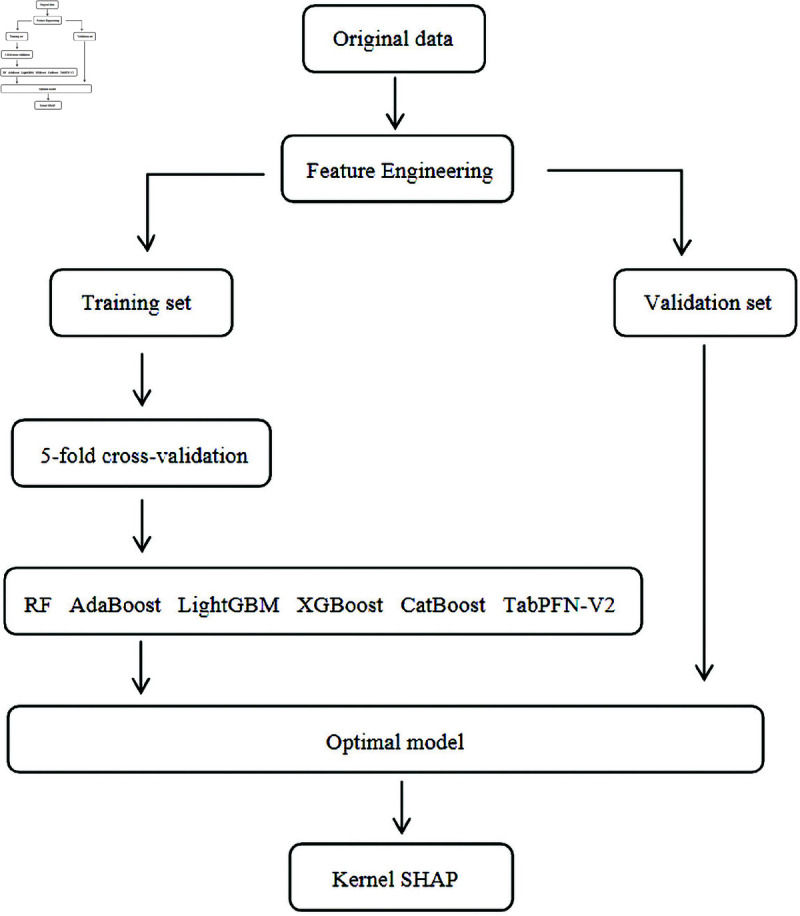
Methodology block diagram.

To ensure robust generalization and mitigate overfitting, all evaluations employed stratified 5-fold cross-validation, with final model selection based on comprehensive validation outcomes across all evaluation criteria (Fig 1). Continuous variables were assessed using the Mann-Whitney *U* test, while categorical variables were evaluated through Pearson’s chi-square test. The *P* value <0.05 was considered statistically significant.

### Data processing methodology

Continuous variables were stratified based on their distributional characteristics. Parameters demonstrating approximate normality (ESR, WBC, N, PLT, Hb, ALB, TP) underwent missing value imputation using arithmetic means. Conversely, non-normally distributed variables (age, CRP, AST, ALT) were imputed with median values. Categorical variables (KDtype, CAL, Recurrence) were completed using mode values. To address class imbalance, this study implemented the Synthetic Minority Over-sampling Technique for Nominal and Continuous features (SMOTE-NC). This algorithm synthesizes new minority class instances through k-nearest neighbor interpolation in feature space, simultaneously preserving categorical variable integrity while augmenting underrepresented categories. The dataset underwent stratified partitioning into training (80%) and validation (20%) subsets, with randomization controlled by a fixed seed (42) to ensure reproducibility. This partitioning strategy maintained proportional class representation across subsets while permitting rigorous evaluation of model generalizability.

### Model explanation

**Fig 2:**
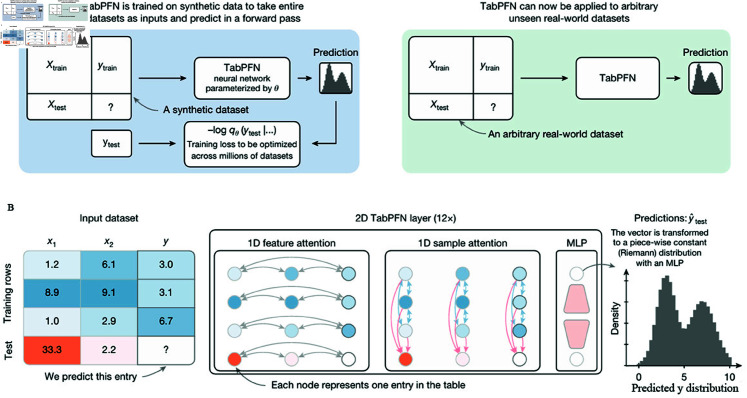
TabPFN model. (A) Overview of TabPFN synthetic pre-training. (B) TabPFN architecture.

The core innovation of TabPFN-V2 lies in its two-stage learning framework: generating extensive synthetic tabular datasets followed by training a Transformer-based architecture to acquire meta-learning capabilities through exposure to diverse synthetic prediction tasks. Unlike conventional approaches that require manual feature engineering or problem-specific adaptations when confronting data challenges like missing values, TabPFN develops intrinsic problem-solving strategies by systematically addressing synthetically generated tasks that emulate real-world data imperfections. This pre-training paradigm enables rapid adaptation to new tabular prediction problems with minimal fine-tuning (Fig 2).

The SHAP 0.42.1 was employed for interpretable ML analysis of the optimal prediction model. Utilizing the kernel SHAP approximation framework, this study conducted both global interpretability analysis and local instance-level explanations. Feature importance ranking was determined by aggregating absolute SHAP values across the dataset, where each feature’s significance was quantified by its mean absolute Shapley value contribution. This integration of ML with game-theoretic interpretation establishes a mathematically grounded framework for explaining predictive model behavior, enhancing both transparency and scientific validity.

## Results

### Clinical cohort characteristics

Table 1 presents the demographic characteristics and laboratory profiles of 1,578 KD patients enrolled in the training and validation cohorts. The study population comprised 937 males (59.4%) with 378 cases (24.0%) exhibiting CALs, 422 cases (26.7%) diagnosed as incomplete KD, and 75 cases (4.8%) experiencing disease relapse. Comparative analysis revealed the IVIG resistant subgroup (n=170, 10.8%) displayed significantly elevated inflammatory markers and hepatic enzymes, including CRP, ESR, N, AST, and ALT. Conversely, this subgroup demonstrated younger age at onset, reduced PLT, and ALB levels compared to IVIG responsive counterparts. Three categorical key predictors of IVIG resistance in KD is Recurrence, CAL, and KDtype (Table 1).

**Table 1:** Comparison of clinical and laboratory characteristics in IVIG responsive and resistant patients.

Variable	Over all (N = 1578)	IVIG responsivenesseak (N = 1408)	IVIG resistance (N = 170)	*P* value
*Malesex*	937	824	113	$0.046^{a}$
*Age*(*mo*)	34.10±25.53	38.30±26.67	33.83±25.58	0.020
*IncompleteKD*	422	391	31	$0.015^{a}$
*CAL*	378	311	67	$<0.001^{a}$
*Recurrence*	75	62	13	$0.007^{a}$
CRP(mg/L)	83.16±68.94	78.54±63.93	123.39±95.47	**0.001**
ESR(mm/h)	58.80±39.79	58.22±40.97	63.91±28.79	**0.004**
WBC(×109/L)	14.46±6.54	14.36±6.32	14.70±5.93	0.393
N(×109/L)	9.55±5.14	9.34±4.96	11.13±5.34	**<0.001**
PLT(×109/L)	351.25±118.67	354.21±117.16	320.15±119.63	**<0.001**
Hb(g/L)	112.62±10.79	112.54±10.64	112.96±11.29	0.601
ALB(g/L)	38.16±5.13	38.47±4.97	36.10±5.63	**<0.001**
AST(U/L)	46.48±83.78	40.42±63.37	79.36±120.10	**<0.001**
ALT(U/L)	52.26±79.79	46.45±69.49	90.64±117.73	**<0.001**
TP(g/L)	66.48±8.32	66.61±7.63	66.47±12.81	0.147

KD, Kawasaki disease; IVIG, intravenous immunoglobulin; CAL, coronary artery lesion; CRP, C-reactive protein; ESR, erythrocyte sedimentation rate; WBC, white blood cell; N, Neutrophil count; PLT, platelet count; Hb, hemoglobin; ALB, albumin; AST, aspartate transaminase; ALT, alanine aminotransferase; TP, total protein. Boldface indicates a statistically significant difference with *P* < 0.05. ^*a*^ Chi-square test.

### Training set accuracy and IVIG responsiveness evaluation

Six ML algorithms (Random Forest, AdaBoost, XGBoost, LightGBM, CatBoost, and TabPFN-V2) were systematically evaluated using 5-fold cross-validation for hyperparameter optimization. The training set accuracies demonstrated notable variation across models: RF achieved 0.82, AdaBoost 0.80, LightGBM 0.84, XGBoost 0.81, CatBoost 0.92, and TabPFN-V2 attained perfect classification accuracy 1.00 (Table 2). This comparative analysis revealed TabPFN-V2 as the top-performing model, exhibiting exceptional predictive capabilities. Further evaluation of the TabPFN-V2 model on the IVIG responsive subgroup demonstrated outstanding classification metrics: precision, recall, and F1-score all reached 0.98. The model’s perfect training accuracy and strong subgroup performance suggest exceptional pattern recognition capabilities on this dataset, though potential overfitting considerations warrant further investigation in validation set.

**Table 2:** Training set accuracy and IVIG responsiveness evaluation.

Model	Training accuracy	Precision	Recall	F1-score
*RF*	0.82	0.83	0.73	0.78
*AdaBoost*	0.80	0.79	0.77	0.78
*LightGBM*	0.84	0.82	0.79	0.80
*XGBoost*	0.81	0.84	0.76	0.80
*CatBoost*	0.92	0.90	0.83	0.86
*TabPFN*–*V*2	1.00	**0.98**	**0.98**	**0.98**

RF, Random Forest, LightGBM, Light Gradient Boosting Machine, XGBoost, eXtreme Gradient Boosting, CatBoost, Categorical Boosting, TabPFN-V2, Tabular Prior-data Fitted Network version 2.0.

### Validation set accuracy and IVIG resistance evaluation

**Fig 3:**
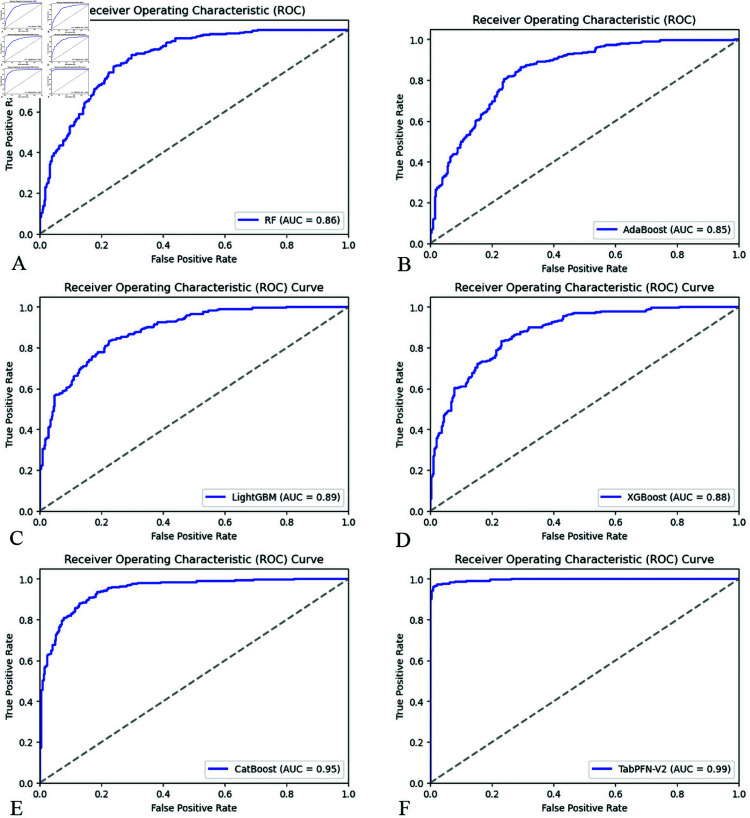
Comparison of AUC for six ML models. (A) Random Forest, (B) AdaBoost, (C) LightGBM, (D) XGBoost, (E) CatBoost, (F) TabPFN-V2.

**Fig 4:**
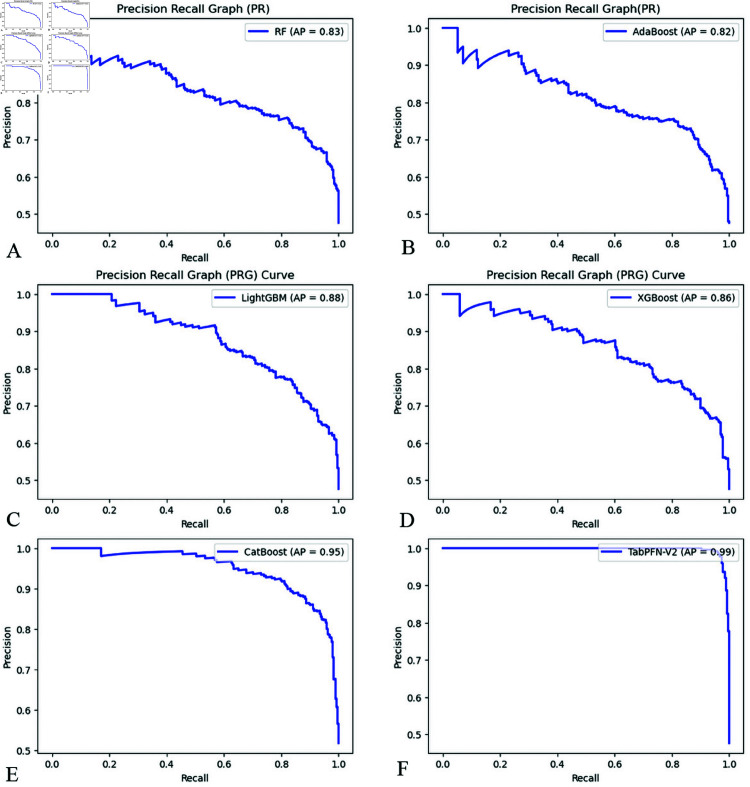
Comparison of PRC for six ML models. (A) Random Forest, (B) AdaBoost, (C) LightGBM, (D) XGBoost, (E) CatBoost, (F) TabPFN-V2.

The validation set was utilized to evaluate the trained models, with subsequent evaluation demonstrating TabPFN-V2’s superior performance. As shown in Table 3, this model achieved the highest accuracy (0.97) while exhibiting optimal model fit without evidence of overfitting. In the IVIG resistance classification, TabPFN-V2 displayed exceptional metrics: precision of 0.98, recall of 0.97, F1 score of 0.98, and MCC of 0.95. The model’s discriminative power was further evidenced by AUC of 0.99 (Fig 3) and PRC of 0.99 (Fig 4). Collectively, these robust performance metrics across multiple evaluation frameworks identify TabPFN-V2 as the superior model for the current dataset. The model demonstrates optimal suitability for classification modeling tasks in this domain, combining high predictive accuracy with stable generalization.

**Table 3:** Training set accuracy and IVIG responsiveness evaluation.

Model	Validation accuracy	Precision	Recall	F1-score	MCC	AUC	PRC
*RF*	0.78	0.74	0.84	0.78	0.57	0.86	0.83
*AdaBoost*	0.77	0.75	0.78	0.77	0.55	0.85	0.82
*LightGBM*	0.79	0.78	0.81	0.79	0.60	0.89	0.88
*XGBoost*	0.79	0.76	0.84	0.80	0.60	0.88	0.86
*CatBoost*	0.87	0.86	0.91	0.88	0.75	0.95	0.95
*TabPFN*–*V*2	**0.97**	**0.98**	**0.97**	**0.98**	**0.95**	**0.99**	**0.99**

RF, Random Forest, LightGBM, Light Gradient Boosting Machine, XGBoost, eXtreme Gradient Boosting, CatBoost, Categorical Boosting, TabPFN-V2, Tabular Prior-data Fitted Network version 2.0.

### TabPFN-V2 model interpretation with kernel SHAP method

**Fig 5:**
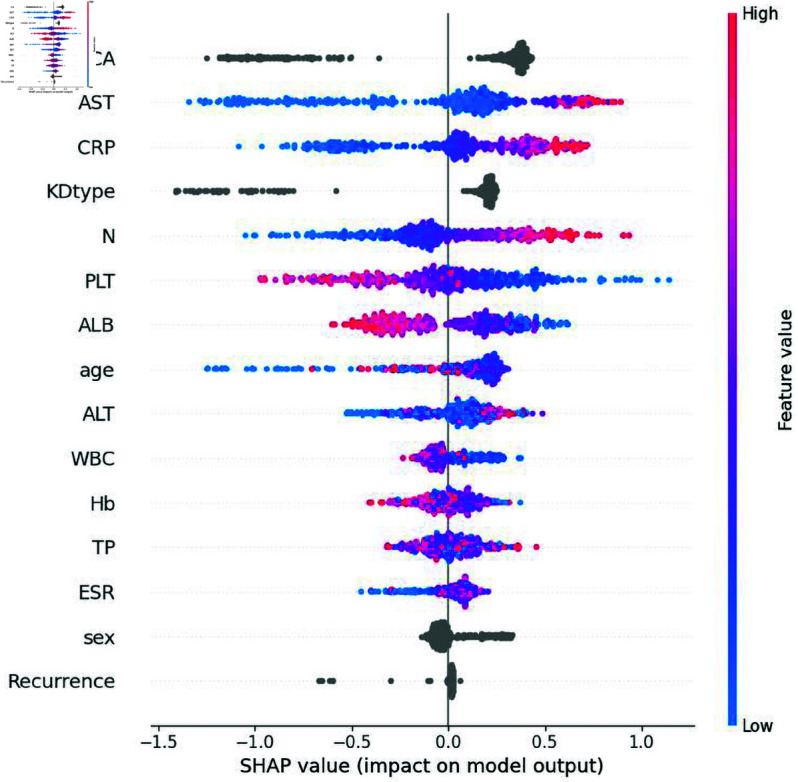
The global interpretability based on kernel SHAP under the TabPFN-V2 model. CA, coronary artery lesions; AST, aspartate transaminase; CRP, C-reactive protein; KDtype, incomplete Kawasaki disease; N, Neutrophil count; PLT, platelet count; ALB, albumin; ALT, alanine aminotransferase; WBC, white blood cell; Hb, hemoglobin; TP, total protein. ESR, erythrocyte sedimentation rate; SHAP, SHapley Additive exPlanations.

The TabPFN-V2 model was analyzed using the kernel SHAP method to quantify feature importance in predicting IVIG resistance. Global interpretability results (Fig 5) reveal the relative influence of clinical variables through distinct horizontal bands, where each feature’s contribution is visualized as color-coded data points across all patient cases. Red and blue markers correspond to high and low SHAP values, respectively, indicating positive and negative impacts on IVIG resistance risk. Among the analyzed predictors, AST, CRP, N, PLT, and ALB emerged as the most influential determinants. Notably, CAL and incomplete KD presentation demonstrated substantial predictive weight. In contrast, patient sex and disease recurrence exhibited minimal contributions to the model’s decision-making framework. This hierarchical feature importance profile provides actionable insights for clinical risk stratification while highlighting key biomarkers warranting closer monitoring.

### Interaction network analysis

**Fig 6:**
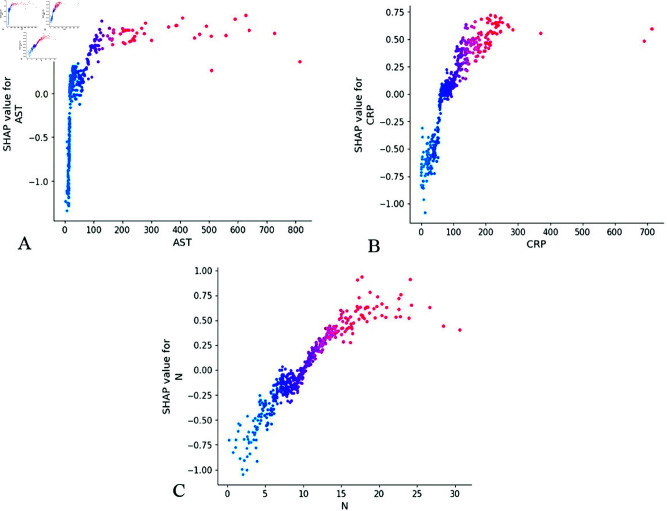
Feature dependence plots. (A) The X-axis represents the range of characteristic values for AST features, and the Y-axis represents the shap value of AST features, which will affect the output of the model. (B) Feature dependence plots of CRP. (C) Feature dependence plots of N. AST, aspartate transaminase; CRP, C-reactive protein; N, Neutrophil count.

In Fig 6, the SHAP scores for AST, CRP, and N demonstrate a positive correlation with IVIG resistance, showing a progressive increase as biomarker values rise. However, this relationship exhibits a non-linear pattern where the contribution value plateaus beyond specific thresholds despite continued biomarker elevation. Notably, the analysis identifies critical inflection points (AST >100 U/L, CRP >110 mg/L, and N >12×10^9^/L) corresponding to SHAP values exceeding baseline (SHAP >0.0). These threshold parameters demonstrate significant predictive value and may serve as foundational criteria for developing a single-index IVIG resistance scoring system.

**Fig 7:**
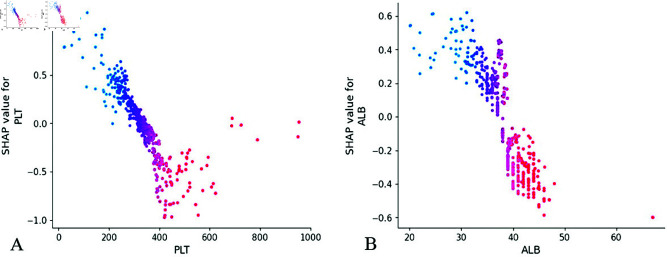
Feature dependence plots. (A) The X-axis represents the range of characteristic values for PLT features, and the Y-axis represents the shap value of PLT features, which will affect the output of the model. (B) Feature dependence plots of ALB. PLT, platelet count; ALB, albumin.

The SHAP dependence plots in Fig 7 reveal a significant negative correlation between PLT and ALB with IVIG resistance risk. As these hematological parameters increase in value, their corresponding SHAP values demonstrate a progressive decline, suggesting diminishing predictive contribution to IVIG resistance at higher measurement ranges. This inverse relationship confirms PLT and ALB as protective factors. Notably, the SHAP threshold analysis identifies clinically relevant cutoff values when the SHAP value crosses below zero. Specifically, PLT<300×10^9^/L and ALB<38 g/L emerge as critical thresholds for elevated IVIG resistance risk. These biomarkers demonstrate particular prognostic value in the subnormal range, potentially serving as key components for developing a weighted prognostic scoring system to stratify IVIG resistance risk.

**Fig 8:**
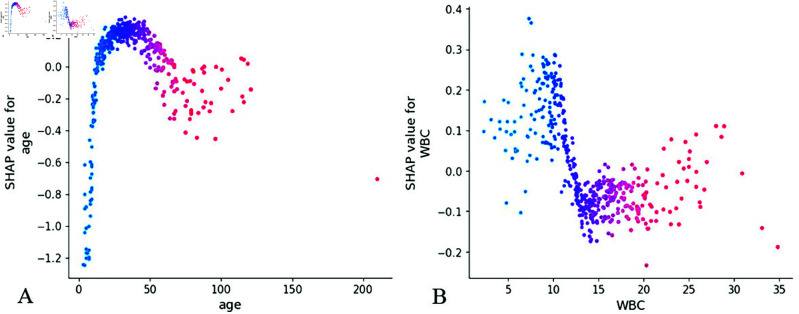
Feature dependence plots. (A) The X-axis represents the range of characteristic values for age features, and the Y-axis represents the shap value of age features, which will affect the output of the model. (B) Feature dependence plots of WBC. WBC, white blood cell.

While variables such as age and WBC in Fig 8 demonstrate relatively lower feature importance compared to other predictors, the SHAP interpretation reveals clinically meaningful value ranges: age between 25-50 months and WBC levels of 12-15×10^9^/L. These identified thresholds offer valuable insights for developing a clinical scoring system, particularly in establishing evidence-based cutoff points for risk stratification.

**Fig 9:**
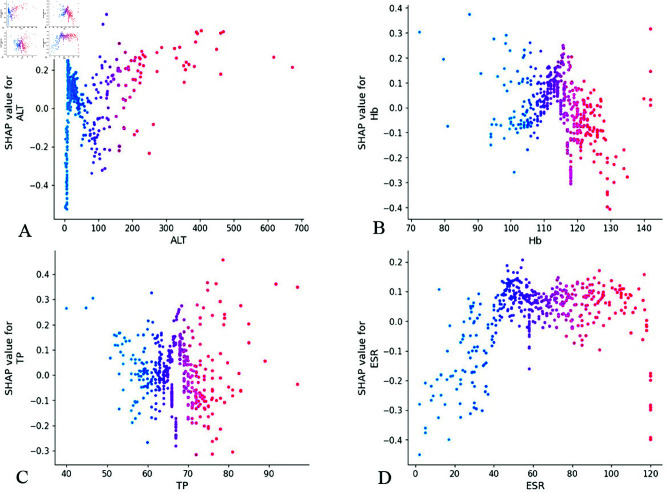
Feature dependence plots. (A) The X-axis represents the range of characteristic values for ALT features, and the Y-axis represents the shap value of ALT features, which will affect the output of the model. (B) Feature dependence plots of Hb. (C) Feature dependence plots of TP. (D) Feature dependence plots of ESR. ALT, alanine aminotransferase; Hb, hemoglobin; TP, total protein. ESR, erythrocyte sedimentation rate.

Conversely, biochemical markers including ALT, Hb, TP, and ESR demonstrated negligible contribution to model performance, suggesting their potential exclusion from future predictive models in similar clinical contexts (Fig 9).

## Discussion

This study developed a predictive model for IVIG resistant KD by employing six ML algorithms to identify critical risk factors. Among the evaluated models (RF, AdaBoost, LightGBM, XGBoost, CatBoost, and TabPFN-V2), the TabPFN-V2 algorithm demonstrated superior predictive performance. SHAP value analysis revealed coronary artery injury and incomplete KD diagnosis as key risk factors for IVIG resistance. Biomarker analysis identified positive correlations between IVIG resistance and elevated levels of AST, CRP, and neutrophil count, with higher values exhibiting greater predictive importance. Conversely, PLT and ALB showed negative correlations, where increased values corresponded to reduced IVIG resistance risk, establishing them as protective factors. Furthermore, threshold optimization for age and WBC parameters enabled refinement of single-variable predictive models, enhancing their clinical applicability.

Existing predictive frameworks for IVIG resistance in KD have demonstrated limited discriminative performance across heterogeneous cohorts [16–18]. ML analysis revealed that the TabPFN-V2 algorithm significantly outperformed conventional models in internal validation, achieving superior predictive accuracy across multiple evaluation metrics including MCC=0.95, AUC=0.99, and PRC=0.99. This advancement enables early identification of high-risk pediatric patients during the critical therapeutic window, potentially facilitating timely escalation to adjuvant therapies such as corticosteroid administration or biologic agents. However, the generalizability of these findings requires verification through multicenter prospective studies with diverse ethnic populations.

The development of TabPFN originates from the inherent limitations of current traditional ML methods in handling tabular data challenges, particularly when dealing with structural heterogeneity across features and inherent complexity in raw data representations. Released in 2025, TabPFN-V2 represents a significant advancement in tabular data processing, expanding its capabilities beyond classification tasks to include native support for regression analysis. This upgraded architecture maintains its intrinsic handling of missing values and outlier detection while eliminating the need for manual feature engineering. Demonstrating superior performance on small-to-medium scale datasets (≤10,000 samples and ≤500 features), the model achieves unprecedented prediction accuracy with dramatically reduced computational requirements. Built upon a generative Transformer foundation, TabPFN-V2 introduces multifunctional capabilities including adaptive fine-tuning, synthetic data generation, probabilistic density estimation, and production of reusable feature embeddings. Its unique training paradigm leverages millions of synthetically generated datasets to develop generalized pattern recognition abilities. Through enhanced cross-domain modeling proficiency, TabPFN-V2 establishes a new paradigm for automated ML, offering transformative potential for accelerating scientific discovery processes and optimizing high-stakes decision-making across industries.

The clinical application of ML models frequently encounters challenges due to their opaque nature and lack of interpretability, raising concerns about their reliability in predicting disease occurrence within medical practice. To address these limitations, this study implemented SHAP value estimation through the TabPFN-V2 architecture and employed kernel SHAP methodology to generate visual explanations of prediction outcomes, subsequently developing a predictive scoring system for IVIG resistance. Prior explainable ML studies furnish a rigorous methodological scaffold and operational protocols that systematically inform IVIG therapeutic optimization research [19].

This investigation identified CAL and incomplete KD as critical predictors of IVIG resistance. The findings suggest that greater inflammatory severity in KD pathogenesis correlates with three clinically significant outcomes: diminished therapeutic response to IVIG, lower diagnostic recognition rates, and increased probability of treatment resistance - particularly in cases with atypical presentations. These insights emphasize the need for enhanced clinical vigilance in detecting incomplete KD manifestations.

The analysis further revealed three key biochemical predictors: elevated AST, CRP, and neutrophil count. These findings align with established pathological mechanisms. First, CRP and neutrophil elevations reflect systemic inflammation consistent with prior studies linking these markers to infectious morbidity [20, 21]. Second, AST elevation specifically indicates cardiovascular compromise associated with CAL. Notably, PLT and ALB emerged as protective factors against IVIG resistance, corroborating conventional diagnostic models [5] though highlighting discrepancies in current scoring systems’ representation of CRP, N, and AST significance. The model optimization identified critical thresholds for risk stratification: age 25-50 months and WBC 12-15×10^9^/L. These parameters demonstrate significant predictive value for IVIG resistance, potentially informing future diagnostic criteria development.The kernel SHAP analysis provides clinically actionable insights. First, Quantifying variable contributions through unified scaling. Second, Enabling transparent interpretation of ML outputs. Third, Facilitating personalized risk assessment beyond generic algorithmic predictions. This explanatory framework enhances clinical decision-making while acknowledging the need for further validation of kernel SHAP’s practical utility in medical contexts.

This study acknowledges several noteworthy limitations that warrant careful consideration. First, while the cohort size exceeds those reported in prior studies, it remains suboptimal relative to the data requirements of modern ML algorithms, potentially limiting model generalizability. Second, the absence of a systematic methodology for feature selection introduces susceptibility to data noise contamination, which may compromise model efficacy through spurious correlations. Third, despite TabPFN-V2’s established versatility in tabular data processing, the single-institution provenance of dataset raises concerns regarding clinical translatability, necessitating rigorous external validation across multi-center cohorts with geographic and demographic diversity. The current study was constrained to fundamental laboratory parameters, while omitting more comprehensive analyses of clinically relevant biomarkers including many liver function markers, Epstein-Barr virus serological profiles, immunological indicators, and echocardiographic parameters. The absence of these multidimensional datasets represents a significant methodological limitation that merits systematic exploration in subsequent research endeavors. Regarding model interpretability, although SHAP analysis was implemented, the computational complexity of kernel SHAP based logical operation interpretation presents substantial challenges in clinical deployment contexts, demanding prohibitive temporal and hardware resources. This underscores the need to investigate emerging interpretability frameworks specifically optimized for medical applications, such as the SHAP-IQ, which may offer pediatricians more clinically actionable insights through enhanced visualization capabilities.

## Conclusion

In conclusion, the findings demonstrate that CAL and incomplete KD presentation serve as significant clinical risk factors for IVIG resistance. Through ML analysis, this study identified elevated AST, CRP, and neutrophil count, along with decreased PLT and ALB levels, as independent predictors of treatment non-responsiveness. The Transformer based TabPFN-V2 prediction model demonstrated superior predictive performance, achieving an AUR of 0.99 in validation cohorts. Through interpretable ML framework using Kernel SHAP analysis, this study confirmed the model’s ability to generate clinically meaningful risk stratification. This advanced decision-support tool holds substantial translational value, enabling early identification of high-risk KD patients and assisting clinicians in optimizing therapeutic strategies through personalized IVIG resistance risk assessment.

## Supporting information

S1 Table TRIPOD+AI checklist for the reporting of prediction model studies (DOC)

S1 Appendix Data and codes are available at: (DOCX)
