# Supplementary material for: Clinical prediction of intravenous immunoglobulin-resistant Kawasaki disease based on interpretable Transformer model
Source: PLoS One. 2025 Jul 9;20(7):e0327564. doi: 10.1371/journal.pone.0327564 (PMC12240358; doi:10.1371/journal.pone.0327564)
Supplement: S1 Appendix — (DOCX) [file pone.0327564.s002.docx]

*[https://github.com/zhuzhuchifei/ kawasaki](https://github.com/zhuzhuchifei/kawasaki)-disease-2025*
